# Circulating tumor cells in early stage lung adenocarcinoma: a case series report and literature review

**DOI:** 10.18632/oncotarget.15506

**Published:** 2017-02-19

**Authors:** Xu-Rui Jin, Lu-Yao Zhu, Kai Qian, Yong-Geng Feng, Jing-Hai Zhou, Ru-Wen Wang, Li Bai, Bo Deng, Naixin Liang, Qun-You Tan

**Affiliations:** ^1^ Department of Thoracic Surgery, Institute of Surgery Research, Daping Hospital, Third Military Medical University, Chongqing 400042, P.R. China; ^2^ Department of Respiratory Disease, Xinqiao Hospital, Third Military Medical University, Chongqing 400037, P.R. China; ^3^ Peking Union Medical College Hospital, Chinese Academy of Medical Sciences and Peking Union Medical College, Beijing 100730, P.R. China

**Keywords:** lung adenocarcinoma, circulating tumor cells, AIS, EMT

## Abstract

**Purpose:**

The study aimed to monitor circulating tumor cells (CTCs) in early stage lung adenocarcinoma patients.

**Results:**

CTCs were characterized and classified to epithelial (E-) CTCs, mesenchymal (M-) CTCs and epithelial- mesenchymal (E&M-) CTCs, as per epithelial-mesenchymal transition(EMT) biomarkers. CTCs could not be found in healthy controls. However, in cohort A, CTCs were found in 17 (17/18) cases. Detection rate of E-CTCs was lower (5/18) compared with M-CTC (10/18) or E&M-CTC (14/18). Highly abundant M-CTCs were prone to being in the tumors > 2 cm. In cohorts A and B, CTCs count increased significantly in all patients with tumor progression (7/7). Higher CTCs level or change range could be found postoperatively in the patients with tumor progression, as compared with patients with disease free survival (*P* < 0.01). Additionally, CTCs detected by CanPatrol^TM^ could be validated by CytoploRare or Pep@MNPs.

**Materials and Methods:**

We included four cohorts of patients and 20 healthy controls. In cohort A, CTCs were detected by a newly established approach, i.e., CanPatrol^TM^, prior to anesthesia and monitored after operation longitudinally. In cohort B, CTCs were not assessed prior to operation, but were longitudinally detected after operation. For validation, we detected FOLR(+)-CTCs by using CytoploRare and EPCAM(+)-CTCs by using Pep@MNPs prior to operation, in cohorts C and D, respectively.

**Conclusion:**

CTCs can be detected in early stage lung adenocarcinoma, even in adenocarcinoma *in situ*, and CTCs detection can effectively monitor tumor progression. The distinguishing of biomarkers of highly invasive and aggressive CTCs warrants further robust study.

## INTRODUCTION

In 2015–2016, 224,390 cases were newly diagnosed with lung cancer in USA [1]. Of all the cases, 83% are non-small cell lung cancer (NSCLC). Currently, the 5-year survival rate of NSCLC patients is 21% [1–3], and more than 25% of early stage NSCLC patients, who have undergone surgical treatment, will have a relapse or progression [1–3].

Circulating tumor cells (CTCs), which shed from the primary tumor into the vasculature or lymphatics, can be regarded as a new prognostic factors of metastatic process [4]. CTCs were exceedingly rare in the blood: one CTC per ~10^7^ white blood cells per milliliter of blood [5–7], hence, extremely sensitive enrichment and detection methods are required to identify and characterize CTCs. Thus far, CTCs-detection technologies can be divided into epithelial cell adhesion molecule (EpCAM)-based detection methods, e.g., the widely used CellSearch^®^ and Adnatest^®^,and EpCAM-independent detection methods, e.g., ISET^®^ and ScreenCell^®^. However, the sensitivities of EpCAM-based detection methods seemed to be significantly lower than EpCAM-independent detection methods [8–11] due to the down-regulation of EpCAM in cancer cells during epithelial-mesenchymal transition (EMT) process.

In stage 1A and 1B lung cancer, the detection of CTCs has found to be a sensitive biomarker to predict the prognosis [12, 13]. In this study, we identified CTCs in early-stage lung adenocarcinoma patients with CanPatrol^TM^ (Surexam Biotech, Guangzhou, China) [14, 15], and explored the subtypes of CTCs as per EMT markers, showing that aberrant activation of EMT could be involved in lung cancer dissemination. Our results showed for the first time that CTCs can be detected in the case with adenocarcinoma *in situ* (AIS) of lung. Additionally, a longitudinal study was performed to assess the clinical implications of continuous monitoring of CTCs. Furthermore, we validated CanPatrol^TM^ by using other two ways to detect Folate receptor (FOLR) (+)-CTCs [16, 17] and EPCAM (+)-CTCs [18], respectively. Finally, we reviewed the published studies regarding the detection of CTCs in a variety of AIS and early stage lung cancer.

## RESULTS

**Figure 1:**
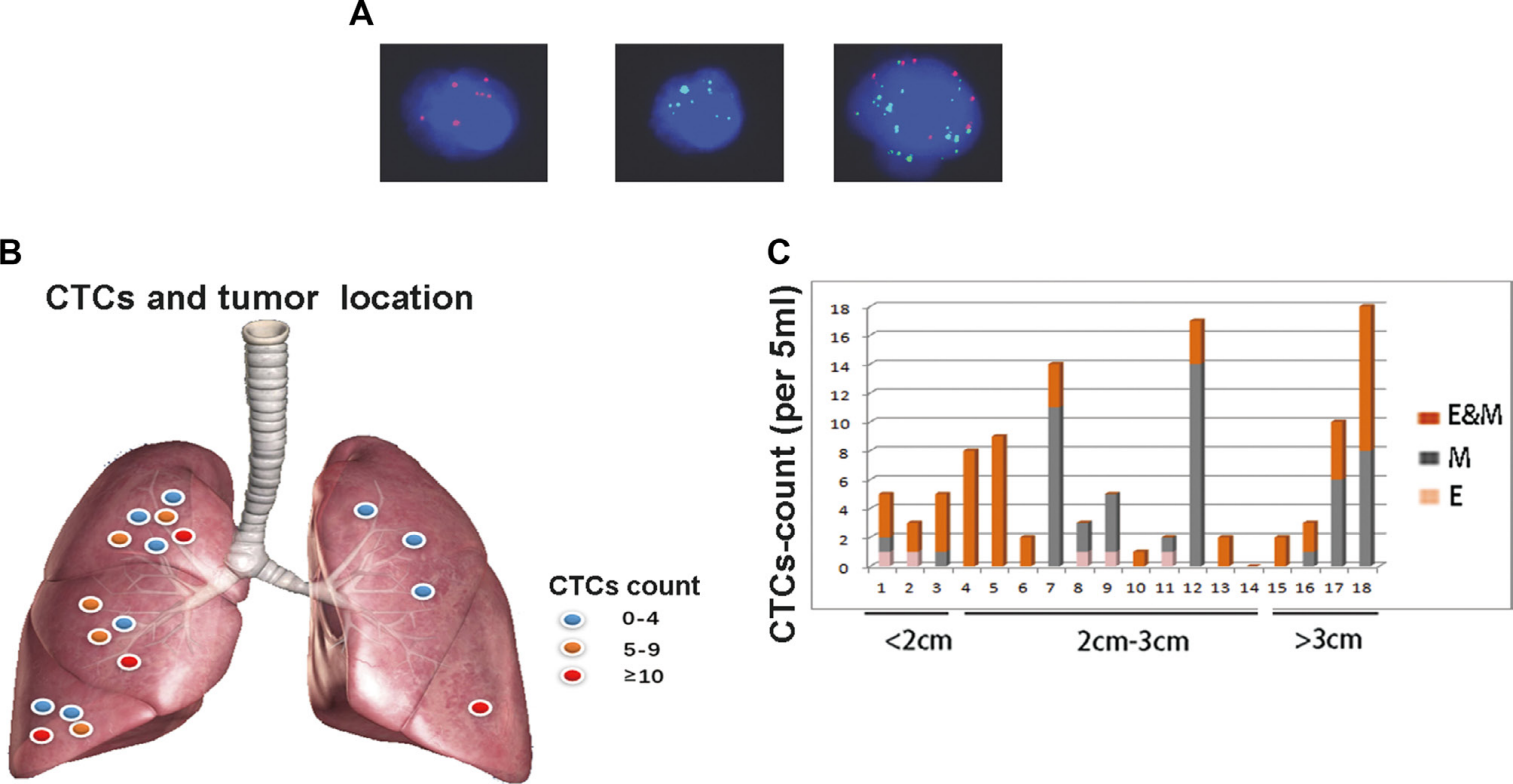
EMT-CTCs prior to operation in cohort A with early stage lung adenocarcinoma (**A**) From left to right, RNA-*in situ* hybridization clearly identified EMT markers in an E-CTC (red dots), a M-CTC (green dots) and an E&M-CTC(red and green dots), respectively. Therefore, EMT-CTCs were classified as E-CTCs, M-CTCs and E&M-CTCs, respectively. (**B**) In the scatter diagram, highly abundant total CTCs (E-CTCs, M-CTCs and E&M-CTCs) was prone to being in the tumors in right side, e.g., case A2(CTCs count = 17), case A10(CTCs count = 14) and case A18(CTCs count = 18). (**C**) Eighteen patients were divided into three groups by tumor size, i.e., < 2 cm, 2cm-3cm and > 3 cm, respectively. The different subtypes of CTCs, i.e., E-, M- and E&M-CTCs were marked by different colors, respectively. M-CTCs were prone to being in the tumors > 2 cm. Furthermore, E-CTCs were prone to being in the tumors < 3 cm.

CTCs could not be found in 20 healthy controls, but were detectable in 17(17/18) cases in cohort A, as shown in Table 1. As shown in Figure 1A, RNA-*in situ* hybridization clearly identified EMT markers in an epithelial (E-) CTC (red dots), a mesenchymal (M-) CTC (green dots) and an epithelial- mesenchymal (E&M-) CTC(red and green dots), respectively. Therefore, these CTCs were classified as E-CTCs, M-CTCs and E&M-CTCs, respectively. In cohort A, E-CTCs, M-CTCs and E&M-CTCs were detected in 5,10 and 14 cases, respectively. There were no statistically significant correlations between the number of CTCs and tumor size, age and gender.

**Table 1:** Clinical and demographic information and CTCs inspection in cases and healthy controls

Cohorts	Cases or healthy controls	Gender	Age	Side	lobe	Tumor Size (cm)	TNM staging^Φ^	Pathology	Status	Follow-up duration^#^ (month)	CTCs count prior to operation			
											E	M	E&M	Total
A (*n =* 18)	Case A1	Female	46	R	M	2	T1aN0M0	IA	DFS	7	0	0	8	8
	Case A2	Male	51	R	M	3	T1bN0M0	LPA	DFS	6	0	14	3	17
	Case A3	Female	59	L	U	3.5	T2aN0M0	IA	Recurrence	6	0	1	2	3
	Case A4	Female	60	R	U	2	T1aN0M0	IA	DFS	7	0	0	9	9
	Case A5	Female	52	L	U	2.5	T1bN0M0	IA	DFS	11	0	0	1	1
	Case A6	Female	53	R	L	0.8	T1aN0M0	AIS	DFS	7	1	1	3	5
	Case A7	Male	64	L	U	1.5	T1aN0M0	PDA	DFS	6	1	0	2	3
	Case A8	Female	41	R	U	2	T1aN0M0	IA	Recurrence	12	0	0	2	2
	Case A9	Female	73	R	U	3	T1bN0M0	IA	DFS	5	0	0	2	2
	Case A10	Female	43	R	L	2	T1aN0M0	IA	DFS	5	0	11	3	14
	Case A11	Female	60	R	L	3.3	T2aN0M0	IA	DFS	5	0	0	2	2
	Case A12	Female	70	R	U	2.2	T1bN0M0	IA	DFS	5	1	4	0	5
	Case A13	Male	50	R	M	2.5	T1bN0M0	IA	DFS	11	1	1	0	2
	Case A14	Male	60	L	L	3.5	T2aN0M0	IA	DFS	10	0	6	4	10
	Case A15	Male	71	R	M	1.9	T1aN0M0	MDA	DFS	10	0	1	4	5
	Case A16	Female	62	R	U	3	T1bN0M0	IA	DFS	9	0	0	0	0
	Case A17	Male	62	R	L	2	T1aN0M0	IA	Death^∮^	10	1	2	0	3
	Case A18	Female	63	R	U	5	T2aN0M0	IA	DFS	12	0	8	10	18
B (*n =* 19)	Case B1	Male	61	R	L	5	T2aN0M0	PDA	Bone metastasis	20	N.A.			
	Case B2	Male	52	R	M	4	T2aN0M0	IA	Recurrence	20	N.A.			
	Case B3	Male	56	R	U	3.2	T2aN0M0	IA	Recurrence	12	N.A.			
	Case B4	Female	60	L	U	3	T1bN0M0	IA	Recurrence	20	N.A.			
	Case B5	Male	56	L	L	3	T1bN0M0	IA	Hepatic metastasis	60	N.A.			
	Case B6	Female	63	L	U	2	T1aN0M0	IA	DFS	36	N.A.			
	Case B7	Female	59	R	U	1.8	T1aN0M0	IA	DFS	12	N.A.			
	Case B8	Female	52	R	U	3	T1bN0M0	IA	DFS	24	N.A.			
	Case B9	Female	49	L	U	3	T1bN0M0	IA	DFS	18	N.A.			
	Case B10	Female	47	L	U	2.5	T1aN0M0	IA	DFS	24	N.A.			
	Case B11	Male	49	R	L	3	T1bN0M0	IA	DFS	12	N.A.			
	Case B12	Female	49	R	U	3	T1bN0M0	IA	DFS	18	N.A.			
	Case B13	Male	53	R	U	2	T1aN0M0	IA	DFS	36	N.A.			
	Case B14	Female	56	R	L	2.5	T1bN0M0	IA	DFS	27	N.A.			
	Case B15	Male	63	R	L	2	T1aN0M0	IA	DFS	12	N.A.			
	Case B16	Male	60	L	U	0.8	T1aN0M0	AIS	DFS	24	N.A.			
	Case B17	Female	51	R	L	2.5	T1bN0M0	IA	DFS	15	N.A.			
	Case B18	Male	59	L	U	2.8	T1bN0M0	IA	DFS	20	N.A.			
	Case B19	Male	55	R	M	3.5	T2aN0M0	IA	DFS	36	N.A.			
C (*n =* 5)	Case C1	Female	77	L	L	3.5	T2aN0M0	IA	DFS	1	1	0	6	7
	Case C2	Male	69	R	U	1.5	T1aN0M0	IA	DFS	1	4	0	2	6
	Case C3	Female	62	R	L	3.1	T2aN0M0	IA	DFS	1	2	0	1	3
	Case C4	Female	64	L	U	2.3	T1bN0M0	MDA	DFS	1	0	0	3	3
	Case C5	Male	68	R	M	2.1	tuberculoma		DFS	1	0	2	3	5
D (*n =* 3)	Case D1	Male	64	L	L	6	T2bN0M0^Δ^	LCNC	DFS	6	1	5	8	14
	Case D2	Male	62	R	U	2.5	T1bN0M0	IA	DFS	6	4	0	2	6
	Case D3	Female	45	R	M	3.5	T2aN0M0^Δ^	IA	DFS	9	0	3	12	15
Healthy controls (*n =* 20)		7 Males 13 females	29.85 (mean age)	N.A.	N.A.	N.A.		N.A.	N.A.	N.A.	0*	0*	0*	0*

Note: IA: invasive adenocarcinoma; DFS: Disease free survival; LPA: lepidic predominant adenocarcinma; LCNC: Large cell neuroendocrine carcinoma;

AIS: adenocarcinoma *in situ*; PDA: poorly-differentiated adenocarcinoma; MDA: moderate-differentiated adenocarcinoma. N.A.: Not Available.

*: We did not find any CTC in 20 healthy controls.

#: Duration from diagnosis to last follow-up or confirmed tumor progression.

Ф: Die of pneumonia

Δ: Visceral pleural invasion

φ:Tumors were staged according to the American Joint Committee on Cancer 2010 Cancer Staging Manual

Figure 1B demonstrated that highly abundant CTCs (total number of E-CTCs, M-CTCs and E&M-CTCs) were prone to being in the tumors in right side, e.g., case A2(CTCs count = 17), case A10(CTCs count = 14) and case A18(CTCs count = 18), although there was no statistical significance difference between right and left side.

As shown in Figure 1C, we divided cohort A into three groups as per tumor size, i.e., < 2 cm, 2–3 cm and > 3 cm, respectively. Intriguingly, E-CTCs were prone to being in the tumors < 3 cm (Figure 1C). Furthermore, highly abundant M-CTCs were prone to being in the tumors > 2 cm (Figure 1C), suggesting these CTCs that have undergone EMT potentially render high risk of metastasis in these cases.

**Figure 2:**
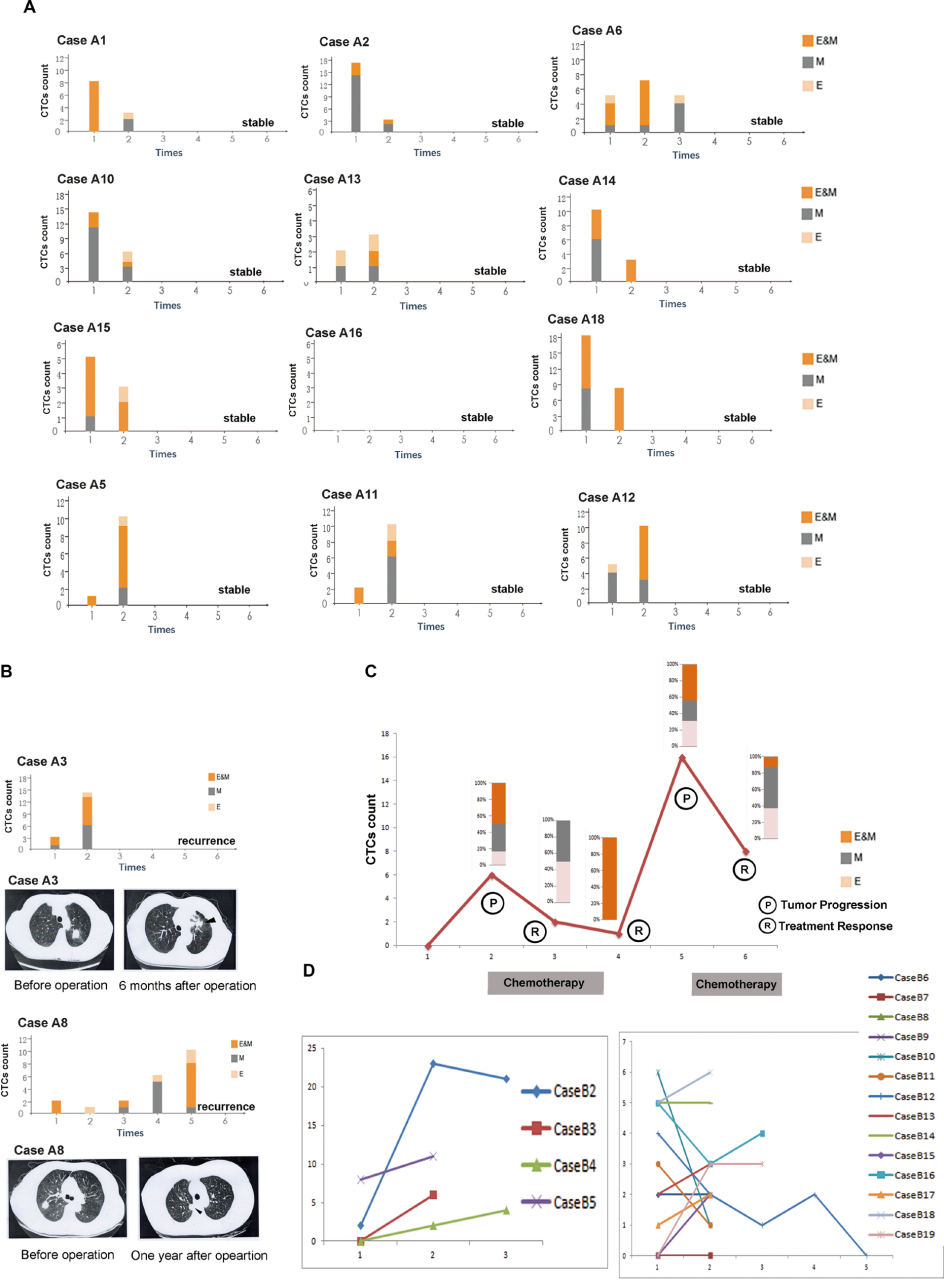
Longitudinal studies of EMT-CTCs in cohorts (**A**) and (**B**). A CTCs monitoring in the stable cases in cohort A. Case A1: 8 CTCs (prior to operation) to 3 CTCs(one month after operation). Case A2: 17 CTCs(prior to operation) to 3 CTCs(one month after operation). Case A6: 5 CTCs (prior to operation), 7 CTCs (four month after operation) to 5 CTCs(one year after operation). Case A10: 14 CTCs (prior to operation) to 6 CTCs(one moth after operation). Case A13: 2 CTCs (prior to operation) to 3 CTCs (one year after operation). Case A14: 10 CTCs(prior to operation) to 3 CTCs (one year after operation). Case A15: 5 CTCs(prior to operation) to 3 CTCs (one year after operation). Case A16: 0 CTC(prior to operation) to 0 CTC (one year after operation). Case A18: 18 CTCs(prior to operation) to 8 CTCs (one year after operation). Case A5: 1 CTCs(prior to operation) to 10 CTCs (one year after operation). Case A11: 2 CTCs(prior to operation) to 10 CTCs (eight months after operation). Case A12 5 CTCs(prior to operation) to 10 CTCs (one year after operation). (B) In case A3, CTCs remarkably increased in six months after operation compared with the preoperative CTCs, and chest CT and biopsy proved the local recurrence (arrow). In case A8, CTCs decreased in six months after the operation, however, increased gradually in eight months, ten months and one year after the operation, and chest CT and biopsy finally proved the local recurrence(arrow). (**C**) In case B1, CTCs count significantly increased from 0 (three months after operation) to 6 (six months after operation). Docetaxel & cisplatin were given to the patient, and CTCs count gradually decreased to 2 (after two cycles of chemotherapy) and 1 (after four cycles of chemotherapy). However, CTCs count increased to 16 and bone metastasis was proved, six months after finished chemotherapy. Therefore, two cycles of Pemetrexed & cisplatin plus Pamidronate Disodium were given to the patient, and CTCs count decreased to 8. (**D**) In cases B2-B5 with tumor progression, CTCs count significantly increased after operation. CTCs count and testing time (months after operation) of each case is present as follows. Case B2: 2 CTCs (eight months), 23 CTCs (thirteen months) to 21 CTCs (eighteen months). Case B3: 0 CTC (two months) to 6 CTCs (seven months). Case B4: 0 CTC (six months), 2 CTCs (one year) to 4 CTCs (sixteen months. Case B5: 8 CTCs (48 months) to 11 CTCs (51 months) In cases B6-B19 with disease free survival, CTCs count significantly decreased in cases B10-B12, slightly increased in cases B8, B9, B13, B15, B17, B18 and B19, or remained stable in cases B6, B7, B14, and B16. CTCs count and testing time (months after operation) of each case is present as follows. Case B6: 2 (18 months) to 2 (22 months). Case B7: 0 (four months) to 0 (nine months). Case B8: 1(13 months) to 2 (21 months). Case B9: 0 (one month) to 2 (three months). Case B10: 6 (20 months) to 1 (24 months). Case B11: 3 (one month) to 1 (four months). Case B12: 4 (two months), 2 (five months), 1(eight months), 2(12 months), to 0 (18 months). Case B13: 2 (24 months) to 3 (36 months). Case B14: 5 (six months) to 5 (nine months). Case B15: 0 (one month) to 2 (six months). Case B16: 5(seven months) to 4 (22 months). Case B17: 1 (one month) to 2 (13 months). Case B18: 5 (13 months) to 6 (16 months). Case B19: 0 (19 months), 3 (26 months) to 3 (35 months).

We performed longitudinal studies in 14 cases of cohort A after operation as shown in Figure 2. Figure 2A showed total CTCs in cases A1, A2,A10,A14,A15 and A18 decreased significantly, ranging from one month to one year after operation, respectively. CTCs count seemed to be stable in case A6 [5 (before operation) to 5 (one year after operation)] and in case A16 [0 (before operation) to 0 (one year after operation)]. In case A13, two CTCs prior to operation slightly increased to three CTCs in one year after operation. Follow-up studies demonstrated disease free survival in the abovementioned nine cases.

However, as compared with before operation, cases A5, A11 and A12 had significant increase of CTCs after operation (Figure 2A), while the values of tumor markers, e.g., CEA, CA125 and CA199 were still normal (data not shown). Careful follow up will be performed among these cases in spite of the current absence of definitive evidence regarding tumor progression.

Cases A3 and A8 respectively had a recurrence in six months and one year after the operation, proved by the increased CTCs (Figure 2B), computed tomography (Figure 2B) and biopsy.

Longitudinal CTCs monitoring was performed in case B1 as shown in Figure 2C, suggesting CTCs count can effectively monitor tumor activity. CTCs count significantly increased from 0 (three months after operation) to 6 (six months after operation). Docetaxel & cisplatin were given to the patient, and CTCs count gradually decreased to 2 after two cycles of chemotherapy and 1 after four cycles of chemotherapy. However, CTCs count increased to 16 and bone metastasis was proved, six months after finished chemotherapy. Therefore, Pemetrexed & cisplatin plus Pamidronate Disodium were given to the patient, and CTCs count decreased to 8 after two cycles of chemotherapy.

In cases B2-B5, CTCs count significantly increased after operation (Figure 2D). Intriguingly, tumor markers were also found to be elevated finally in case B2 [CA125: 81.07 U/ml (normal range: 0–35)], case B3 [CEA: 5.85 ng/ml (normal range: 0–5); CA125: 179.14 U/ml (normal range: 0–35)] and case B4 [CEA: 20.85 ng/ml (normal range: 0–5); CA125: 50.07 U/ml( normal range: 0–35)], and all decreased to normal level after EGFR-tyrosine kinase inhibitor treatment. In case B5, CTCs remained high (8 and 11), and hepatic metastasis was proved.

In cases B6-B19 with disease free survival, CTCs count significantly decreased in cases B10-B12, slightly increased in cases B8, B9, B13, B15, B17, B18 and B19, or remained stable in cases B6, B7, B14, and B16, as shown in Figure 2D. Totally, patients with tumor progression had remarkably higher CTCs level after operation as compared with patients with disease free survival (cases B1-B5 vs. cases B6-B19: 11.00 ± 8.52 vs. 3.21 ± 1.76, *P <* 0.01). Furthermore, change range of CTCs in patients with tumor progression was significantly higher as compared to patients with disease free survival (cases B1-B5 vs. cases B6-B19: 10.00 ± 8.03 vs. −0.21 ± 2.26, *P <* 0.01).

**Figure 3:**
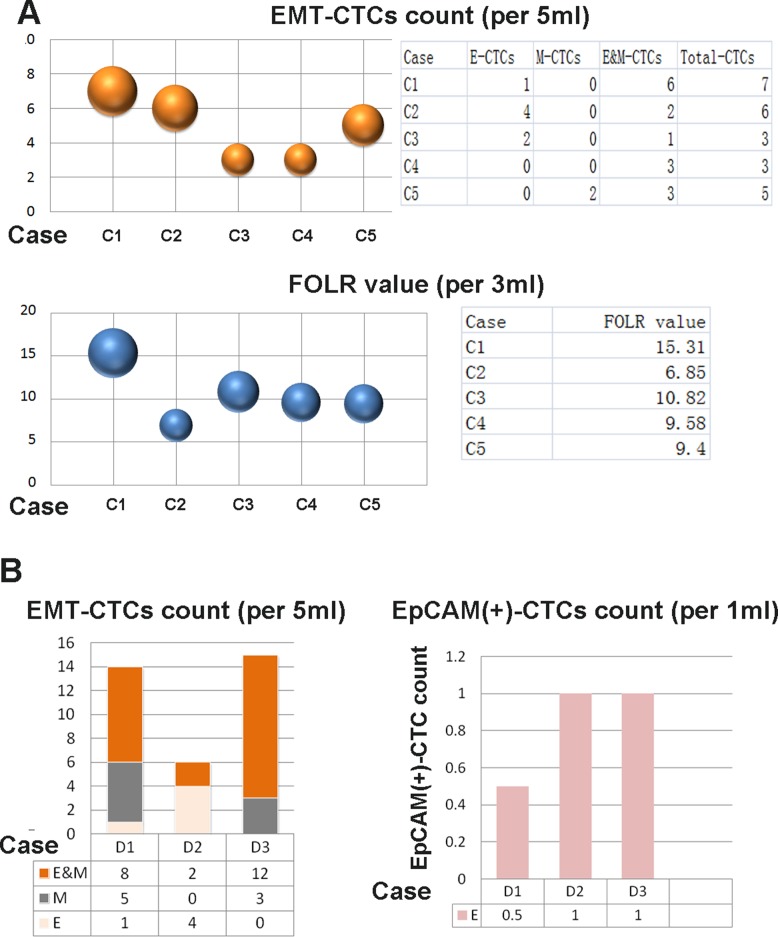
Detection of CTCs by using different methods We entitled the detected CTCs by CanPatrol^TM^, CytoploRare and Pep@MNPs, as EMT-CTCs, (FOLR)(+)-CTCs and EPCAM(+)-CTCs, respectively. (**A**) EMT-CTCs and FOLR(+)-CTCs were simultaneously detected prior to operation in cases C1-C5. EMT-CTCs were detectable in cases C1-C5, and FOLR(+)-CTCs were positive (FOLR value > 8.7 FU/3 ml) in cases C1, C3, C4 and C5. The size of balls represented the CTCs count or FOLR value. (**B**) EMT-CTCs and EPCAM(+)-CTCs were simultaneously detected prior to operation in cases D1-D3 E-CTCs were detectable in cases D1 and D2, and EpCAM(+)-CTCs were positive in cases D1–D3, suggesting Pep@MNPs probably is more capable of capturing epithelial CTCs.

For distinguishing, we entitled the detected CTCs by CanPatrol^TM^, CytoploRare and Pep@MNPs, as EMT-CTCs, (FOLR)(+)-CTCs and EPCAM(+)-CTCs, respectively. As shown in Figure 3A, EMT-CTCs and FOLR(+)-CTCs were simultaneously detected prior to operation in cohort C. In cases C1-C4 with lung adenocarcinoma EMT-CTCs were detectable (4/4), and FOLR(+)-CTCs were positive (FOLR value > 8.7 FU/3 ml) in three cases (C1, C3 and C4). Intriguingly, both EMT-CTCs and FOLR(+)-CTCs were found in case C5 with tuberculoma. As shown in Figure 3B, EMT-CTCs and EPCAM(+)-CTCs were simultaneously detected prior to operation in cases D1-D3 with lung cancer. Both EMT-CTCs and EpCAM(+)-CTCs were positive in cases D1-D3. However, in case D3, E-CTCs detected by CanPatrol^TM^ were not detectable but EpCAM(+)-CTCs were positive, suggesting Pep@MNPs probably is more capable of capturing epithelial CTCs. Totally, our results demonstrated CTCs detected by CanPatrol^TM^ can be validated by CytoploRare or Pep@MNPs in lung cancer patients.

## DISCUSSION

CTCs can be found in early stage of tumors, even in a variety of AIS, e.g., breast cancer *in situ*, melanoma *in situ* and bladder cancer *in situ* (Online Table 2). In our study, CTCs were detectable before or after operation in cases A6 and B16 with lung AIS. Interestingly, CTCs were found in 5 COPD patients without clinically detectable lung cancer [19], and all of them developed into cancer in 1 to 4 years, suggesting CTCs may predict the progression of lung cancer in COPD cases [19].

**Table 2:** CTCs in the cases with carcinoma *in situ*

PMID	Tumor type	Case(*n*)	Test Time	Cell isolation/ confirmation	Marker	CTC	Prognosis
21264346	breast cancer *In situ*	73	3wto 5y after surgery	Cell Search^®^	EpCAMHER2	Positive in 4.1%patients	N.A.
21207426	breast cancer *In situ*	30	At the time of surgery before tumor excision	Immuno-cyto-chemical assay	CKs	DTCs-positive in 21.1% patients	DTC+ patients had Relapse or metastasis
20535130	melanoma *in situ*	17	Pre-operatively	ISET (ScreenCell^®^)/RT-PCR	Tyro-sinase	0%	N.A.
20651396	breast cancer *in situ*	12	At the time of diagnosis	Ficoll-Hipaque (Biochrome AG, Germany)/RT-PCR	hMAM	0	N.A.
22351740	bladder cancer *In situ*	8	At the first time of diagnosis	Cell Search^®^	EpCAM CKs	Positive in 62.5%patients	CTC+ cases: shorter time to recurrence
23088337	breast cancer Insitu	48	Pre-operatively	Cell Search^®^	EpCAM CKs	Positive in 18.7% patients	N.A.

Note: CKs: cytokeratins; DTCs: disseminated tumor cells in bone marrow; EpCAM: epithelial cell adhesion molecule; HER2: human epidermal growth factor receptor-2; hMAM: human mammaglobin; N.A. not addressed.

CTCs had been reported to be detectable in early stage NSCLC (Online Table 3). However, another study demonstrated CTCs test had insufficient capability of discrimination between lung cancer and nonmalignant diseases, although CTC counts were significantly higher in lung cancer patients than in nonmalignant patients [20]. Indeed, both EMT-CTCs and FOLR(+)-CTCs were detectable in a case with tuberculoma as shown in Table 1.

**Table 3:** CTCs in early stage lung cancer

PMID	Staging	Case(n)	Test Time	Cell isolation/ confirmation	Marker	CTC+ Rate	Prognosis
21098695	1A&1B	86	Preoperatively	ISET	Circulating non-hematologic cells	19.4% (7/36 Stage IA) & 28.0% (14/50 Stage 1B)	high risk factor of recurrence and death
26913536	1A&1B	26	Preoperatively	MACS technology	CKsEGFR	50% (13/26)	CTCs after surgery: early recurrence
17554991	1A&1B	9	Preoperatively	Cell Search^®^	CKsEpCAM	16.67% (1/6 Stage 1A) & 0% (0/3 Stage 1B)	N.A.
21128227	Stage 1	91	Preoperatively	Cell Search^®^& ISET	CKsEpCAM	13% (33/91)	CTCs(+): worse DFS
18514066	Stage 1	8	N.A	RT-PCR for Peripheral blood mononuclear cells	BJ-TSA-9,Pre-proGRP, SCC, LUNX, KRT-19	75% (6/8)With at least one marker	Positivity of more than one marker :short survival time
10788810	Stage 1	29	Preoperatively	RT-PCR	Carcinoembryonic antigen (CEA)	37.9 3% (11/29)	Persistently positive CEA mRNA: shorter DFS and OS
23861795	Stage 1	43	1 day after PET	immunofluorescence	CKs	60.47% (26/43)	N.A.
20471712	Stage 1	33	Preoperatively and/or one month after surgery	nested or semi-nested RT-PCR	CK19 and TTF-1	CK19 (+) CTCs: 39.39% (13/33); TTF-1 (+) CTCs:36.4% (12/33)	Postoperative TTF-1(+) CTCs: shorter PFS
25996878	Stage 1	14	Before the treatment	Cell Search^®^& Luminex xMAP assay	CKs	0% (0/14)	N.A
18606477	Stage 1	13	Preoperatively	RT-PCR & ELISA	Survivin	15.38%(2/13)	independent predictor for cancer recurrence
21215651	Stage 1	75	Immediately after pulmonary resection	Immuno-histochemistry	CKs	36% (27 with singular tumor cell)30% (25 with clustered tumor cells)	CTCs: shorter DFS
26317979	Stage 1and 2	16	N.A.	Multi-parameter flow cytometry	EpCAM, CK7/8,CXCR4, CD133	81.25% (13/16)	N.A

Note:CEA: carcinoembryonic antigen;CKs: cytokeratins; CXCR4: C-X-C chemokine receptor type *4;* DFS: disease free survival; EGFR: epidermal growth factor receptor; GRP: gastric inhibition polypeptide; KRT-19: keratin 19; LUNX: lung-specific X protein; PET: positron emission computed tomography; SCC: squamous cell carcinoma antigen; TSA: tumor specific antigen; TTF: thyroid transcription factor.

The diagnostic value of CTCs detection could be enhanced when combined with tumor markers, e.g., CEA, Ki-67, CA125, CA199, Cyfra21-1, and SCCA [21]. In our study, both CTCs count and tumor markers remarkably increased after operation in cases B2-B4, prior to appearance of radiographic evidence of tumor progression. Furthermore, more advanced technologies for CTCs enrichment and detection with high specificity and sensitivity warrant further study.

Currently, technologies for CTCs detection can be divided into EpCAM-based and EpCAM-independent methods, respectively. However, the sensitivity of EpCAM-based detection methods seemed to be relatively low ranging from 21% to 41% [8, 9], showing EpCAM (−) CTCs might be missed in that case. Indeed, EpCAM-independent detection methods rendered high sensitivity ranging from 50% to 100% [10, 11]. In the study, we used the newly established technique, i.e., CanPatrol^TM^, to detect EpCAM, cytokeratins(CKs), vimentin and twist. EpCAM and CKs are commonly expressed in CTCs from epithelial-derived malignancies [14, 15]. Vimentin, a member of the intermediate filament family of proteins, is ubiquitously expressed in mesenchymal cells [14, 15]. Twist, a key transcription factor for EMT, can promote invasion and metastasis, and confer tumor cells with cancer stem cell (CSC)-like characteristics [22]. In the present study, we clustered the CTCs by the abovementioned EMT markers, and found that detection rate of E-CTCs was lower (27.8%, 5/18) compared with M-CTC(55.6%, 10/18) or E&M-CTC(77.8%, 14/18). Indeed, the detection rate of CTCs by using Cellsearch^®^ was especially lower, compared with ISET^®^ as shown in Online Table 3, indicating E-CTCs might not be the main CTC subtype in early stage NSCLC. Furthermore, highly abundant M-CTCs were prone to being in T1b cases, compared with T1a cases. Indeed, compared with E-CTCs, M-CTCs had been found to be more invasive and aggressive, and have the closer correlation with tumor progression [23]. Some newly found biomarkers can increase the positive rate of CTCs detection. Man et al. [24] found that cytokeratin 7(CK7), Ca2+-activated chloride channel-2(CLCA2), hyaluronan-mediated motility receptor(HMMR), and human telomerase catalytic subunit(hTERT) could be detected in 74.0% of 254 lung cancer patients and present a reliable prognosis value. In addition, Folate receptor-positive CTCs showed a sensitivity of 72.5%-73.2% and a specificity of 84.1%-88.7% in the diagnosis of NSCLC, especially a sensitivity of 67.2% in stage I disease [16, 17]. In our study, we tried three methods to detect CTCs in a small series of cases, and found CanPatrol^TM^ can be validated by CytoploRare or Pep@MNPs in lung cancer patients. However, the sensitivity and specificity of CTCs detected by these methods warrant further study.

After surgical removal of tumor mass, some early-stage NSCLC patients will relapse or develop metastases finally. It emphasizes the importance of risk assessment of tumor progression in these patients, and CTCs are considered as the predictive biomarker for guiding cancer treatment strategies [25]. CTCs count was proved to predict the radiation therapy response of NSCLC patients [26]. With regards to the role as a prognostic and predictive factor during chemotherapy, the value of CTCs detection is still inconclusive [27–29]. In NSCLC patients, numerous studies concluded that preoperative or postoperative high CTCs count suggested the shorter lifetime of patients than those with low count [11, 12, 30] and EGFR+ CTCs could predict the high risk of early recurrence after operation [31]. Interestingly, two studies [32, 33] used CellSearch^®^ system to detect CTCs before and after surgery, and found no statistical correlation between EpCAM (+) CTCs count and survival or recurrence, demonstrating EpCAM (−) CTCs or M-CTCs also should be evaluated to monitor tumor progression. In our longitudinal study, CTCs decreased significantly after operation, probably due to the reduced tumor burden, and CTCs significantly increased in all the cases (6/6) of cohorts A and B with tumor relapse or progression, demonstrating CTCs detection can effectively monitor tumor activity.

Theoretically and empirically, CTCs can be erased by anoikis, i.e., a form of programmed cell death that occurs in anchorage-dependent cells when they detach from the surrounding extracellular matrix [34]. As shown in Figure 2A, the number of CTCs in cases A2, A10, A14, A18 before operation was relatively high but decreased remarkably after operation, and the cases were stable after operation. We postulated these CTCs in the above-mentioned cases had been eliminated and formed no tumorous lesion. However, in cases A3 and A8, some critical signaling pathways in CTCs had been activated to help the tumor cells escape from anoikis, leading to tumor recurrence. Indeed, our pilot study had found highly expressed BCAR1, i.e., one of the Crk-associated substrate (cas) protein family members [35] in CTCs in cases A3 and A8(data not shown). Furthermore, our previous studies indicated serum BCAR1 levels were significantly higher in lung cancer compared with the control group, gradually increasing with the progression of tumor staging, and decreasing following malignant lesion removal [36]. In a cohort of 151 Chinese patients with NSCLC, elevated BCAR1 protein expression levels in tumor tissues were shown to predict a poor prognosis [37, 38]. In addition, BCAR1 was found to be required for TGF-beta1-mediated EMT in lung cancer, and BCAR1-knockdown caused cell migration inhibition and arrest of cell growth and the cell cycle in lung cancer cells [35, 37]. Importantly, BCAR1 degradation had been proved to lead to anoikis of cancer cells *in vitro* [39]. Whether these BCAR1(+)-CTCs are more invasive and aggressive than BCAR1(−)-CTCs warrant further robust study.

## CONCLUSION

Collectively, CTCs could not be found in 20 healthy controls, but were preoperatively found in 94% (17/18) cases with lung adenocarcinoma in early stage. E-CTCs, M-CTCs and E&M-CTCs were detected in 27.8% (5/18), 55.5% (10/18) and 77.8 (14/18) cases, respectively. Highly abundant M-CTCs were prone to being in the tumor ≥ 2 cm, compared with tumors < 2 cm, suggesting T1b lung adenocarcinoma may be prone to shedding of highly invasive and aggressive CTCs. Additionally, CTCs detection could effectively monitor tumor progression. Our results demonstrated CanPatrol^TM^ can be validated by CytoploRare or Pep@MNPs in lung cancer patients. The distinguishing of biomarkers of highly invasive and aggressive CTCs warrants further robust study.

## MATERIALS AND METHODS

### Clinical-demographical characteristics

From January 2013 to August 2016, four cohorts of patients (From Daping hospital: cohorts A, C and cases B1-B4 and cases B6-B12 in cohort B; From Peking union medical college hospital: cohort D and case B5 and cases B13-B19 in cohort B) and 20 healthy controls (non-smokers) were enrolled, respectively as shown in Table 1. Informed consent was written by each patient or control who participated this research.

Cohorts A and B included 18 and 19 patients with stage I lung adenocarcinoma, respectively (Table 1). In cohort A, CTCs had been detected prior to anesthesia in all 18 cases. Among them, one patient died of pneumonia and three patients denied re-evaluation of CTCs after operation, hence, CTCs were monitored after operation in 14 cases. In cohort B, CTCs were not assessed prior to operation, but were longitudinally detected after operation.

To distinguish the detected CTCs by using CanPatrol^TM^, CytoploRare and Pep@MNPs, we entitled them as EMT-CTCs, (FOLR)(+)-CTCs and EPCAM(+)-CTCs, respectively. In cohorts C and D, we simultaneously detected EMT-CTCs&FOLR(+)-CTCs and EMT-CTCs&EPCAM(+)-CTCs prior to operation, respectively. In addition, EMT-CTCs had been detected in the healthy controls.

All the cases, except Case A10 who had CT-guided percutaneous lung biopsy prior to operation, did not undergo invasive diagnostic procedure. All the cases underwent video-assisted thoracoscopic surgery(VATS). i.e., lobectomy and lymphadenectomy. The diagnosis was confirmed pathologically in all the cases. The clinical and demographical characteristics were shown in Table 1.

### Detection of EMT-CTCs by using CanPatrol^TM^

Ten ml of blood was collected from the healthy controls and cases, and transferred into sample preservative tubes (Surexam Biotech, Guangzhou, China) containing ammonium chloride-based lysing buffer by a tailored connection device (Surexam Biotech, Guangzhou, China) and incubated at room temperature for 30 min.

CanPatrol ^TM^ was used to detect EMT-CTCs, which is a newly established technology to detect CTCs, containing the following steps [14, 15]: (1) To remove erythrocytes by red blood cell lysis and deplete CD45+ leukocytes in 10 ml blood sample using a magnetic bead separation method; (2) To enrich CTCs by 8-μm-diameter-pore calibrated membrane filters; and (3) To identify and characterize CTCs by using RNA-*in situ* hybridization (ISH), based on the branched DNA (bDNA) signal amplification technology, to detect EMT markers, e.g., cytokeratins(CK) 8, 18 and 19, epithelial cell adhesion molecule (EpCAM), vimentin and twist.

The details of classification of EMT-CTCs by using CanPatrol ^TM^ was depicted in the recently published protocol [40]. Finally, the EMT-CTCs were clustered into three subtypes, as per the EMT markers, i.e., epithelial (E-) CTCs, mesenchymal (M-) CTCs and epithelial- mesenchymal (E&M-) CTCs.

### Detection of FOLR(+)-CTCs by using CytoploRare method

FOLR(+)-CTCs were detected by using CytoploRare method provided by GenoSaber Biotech Co. Ltd. (Shanghai, China) [16, 17]. Blood sample (3 ml) were collected in vacuum tubes containing the anticoagulant ethylenediaminetetraacetic acid, and CTC analysis was performed within one hour.

Firstly, erythrocytes were removed by red blood cell lysis and CD45+ leukocytes were depleted by using a magnetic bead separation method. Then it was labeled with a conjugate of a tumor-specific ligand folic acid and a synthesized oligonucleotide [16, 17]. Thereafter, the CTCs were collected for quantitative PCR analysis. Before amplification, the conjugate first annealed and extended on the reverse transcriptase primer. After immunofluorescence staining the enriched CTCs, FOLR(+)-CTCs were defined as cells expressing folate ligands and cytokeratin and 4′,6-diamidino- 2-phenylindole–stained nucleus.

In this study, we used an arbitrarily defined CTC unit, which was defined as the number of CTCs detected in 3 ml of blood. FOLR value > 8.7 FU/3 ml was defined as positive [16, 17].

### Isolation of EpCAM(+)–CTCs by using Pep^@^ MNPs

EpCAM(+)–CTCs were isolated by using the EpCAM recognition peptide functionalized iron oxide magnetic nanoparticles (MNPs) (Pep@MNPs) [18]. 10 μLof the obtained anti-EpCAM@MNPs was added to 1 mL human blood in a 1.5 mL centrifuge tube. After incubation of the mixed suspension in a shaker at 37°C for 30 min (the optimal conditions), the captured cells were gently washed with PBS at least 5 times under a high magnetic field (116 mT). In order to confirm the cell type of the captured cells from blood samples, the commonly used three-color immunocytochemistry method was applied. The captured cell samples were incubated with FITC-labeled anti-CD45 and APC-labeled anti-CK for 30 min respectively and followed by PBS washing at least 3 times. The CTCs confirmation was performed using a fluorescence microscope.
